# An overview of the regulation of influenza vaccines in the United States

**DOI:** 10.1111/irv.12383

**Published:** 2016-03-24

**Authors:** Jerry P. Weir, Marion F. Gruber

**Affiliations:** ^1^ Laboratory of DNA Viruses/Division of Viral Products/Office of Vaccines Research and ReviewCenter for Biologics Evaluations and ResearchFood and Drug AdministrationSilver SpringMDUSA; ^2^ Office of Vaccines Research and ReviewCenter for Biologics Evaluations and ResearchFood and Drug AdministrationSilver SpringMDUSA

**Keywords:** influenza vaccines, vaccine regulation, vaccine licensing

## Abstract

Influenza virus vaccines are unique among currently licensed viral vaccines. The vaccines designed to protect against seasonal influenza illness must be updated periodically in an effort to match the vaccine strain with currently circulating viruses, and the vaccine manufacturing timeline includes multiple, overlapping processes with a very limited amount of flexibility. In the United States (U.S.), over 150 million doses of seasonal trivalent and quadrivalent vaccine are produced annually, a mammoth effort, particularly in the context of a vaccine with components that usually change on a yearly basis. In addition, emergence of an influenza virus containing an HA subtype that has not recently circulated in humans is an ever present possibility. Recently, pandemic influenza vaccines have been licensed, and the pathways for licensure of pandemic vaccines and subsequent strain updating have been defined. Thus, there are formidable challenges for the regulation of currently licensed influenza vaccines, as well as for the regulation of influenza vaccines under development. This review describes the process of licensing influenza vaccines in the U.S., the process and steps involved in the annual updating of seasonal influenza vaccines, and some recent experiences and regulatory challenges faced in development and evaluation of novel influenza vaccines.

## Introduction

Influenza virus vaccines are unique among currently licensed viral vaccines. Due to the constant antigenic drift of the influenza virus hemagglutinin (HA) and neuraminidase (NA) surface glycoproteins, the vaccines designed to protect against influenza illness must be updated periodically in an effort to match the vaccine strain with the wild‐type viruses circulating in a particular season. Licensed inactivated influenza vaccines have been available in the United States (U.S.) since 1945^1^; a live attenuated influenza vaccine has been available since licensure in 2003.^2^ A list of current U.S. licensed influenza vaccines can be found at http://www.fda.gov/BiologicsBloodVaccines/Vaccines/ApprovedProducts/ucm093830.htm and http://www.cdc.gov/flu/protect/vaccine/vaccines.htm.

In addition to the gradual but continuous antigenic drift of influenza viruses, emergence of an influenza virus containing an HA subtype that has not recently circulated in humans is an ever present possibility. Adequate preparation for an influenza pandemic that would result from such an antigenic shift necessitates the development and evaluation of pandemic influenza vaccines against virus strains that are not currently circulating in the human population. Finally, protection afforded by current seasonal influenza vaccines is not ideal with vaccine effectiveness estimates of approximately 60% for the overall population when the vaccine is well matched to circulating viruses, but with substantially reduced effectiveness when there is a poor match and in certain populations such as the elderly (see http://www.cdc.gov/flu/about/qa/vaccineeffect.htm and references included). Thus, there are formidable challenges for the regulation of currently licensed influenza vaccines, as well as for the regulation of influenza vaccines under development. The current review describes the process of licensing influenza vaccines in the United States, the process and steps involved in the annual updating of seasonal influenza vaccines, and some recent experiences and regulatory challenges faced in development and evaluation of novel influenza vaccines.

## Licensing of seasonal influenza vaccines

The Food and Drug Administration (FDA) is responsible for regulating vaccines in the United States. Licensure of seasonal influenza vaccines follows the same general approach as licensure of other vaccines and has been described previously.^3^ Licensure may be obtained either through a traditional approval pathway or by the accelerated approval mechanism. Both pathways share similar requirements for demonstration of product safety and consistency of vaccine manufacturing. Traditional approval provides pre‐licensure evidence of efficacy from clinical trials in which influenza illness is assessed as the primary endpoint. Recent examples of influenza vaccines that have been approved by the traditional pathway include Flucelvax^®^ in 2012, an inactivated trivalent vaccine produced in cell culture by Novartis Vaccines and Diagnostics, Inc. (in 2015, Novartis influenza vaccines and bioCSL were joined to create Seqirus), and Flublok^®^(Protein Sciences Corp.) in 2013, a recombinant protein vaccine manufactured by Protein Sciences Corporation.

Evaluation of the vaccine‐induced immune response in the clinical disease endpoint efficacy studies is important to potentially extrapolate vaccine effectiveness to populations not included in the efficacy trial. For example, persons 6–59 months of age and those 65 years of age and older may not have been included in efficacy studies because of ethical concerns related to conducting placebo‐controlled efficacy studies in populations for which influenza vaccines are recommended. In such populations, effectiveness can be based on immunogenicity endpoints.^3^

Accelerated approval, on the other hand, is based on adequate and well‐controlled clinical trials establishing that the vaccine has an effect on a surrogate endpoint that is reasonably likely to predict clinical benefit. The FDA's Center for Biologics Evaluation and Research (CBER) considers the hemagglutination inhibition (HI) antibody response an acceptable surrogate marker that is reasonably likely to predict clinical benefit of inactivated influenza vaccines.^3^ Approval under this pathway is subject to the post‐marketing requirement that the sponsor conduct adequate and well‐controlled clinical studies to verify and describe the clinical benefit of the vaccine, that is, protection from influenza disease. Because influenza is a serious and sometimes life‐threatening illness, FDA has interpreted the accelerated approval regulation as allowing accelerated approval of an inactivated influenza vaccine when the supply of influenza vaccine is insufficient to immunize all persons recommended for annual influenza vaccination by the Centers for Disease Control and Prevention (CDC). The accelerated approval regulatory mechanism was used to license the trivalent inactivated influenza vaccines Fluarix^®^ (GlaxoSmithKline Biologicals) in 2005, FluLaval^®^ (ID Biomedical Corporation of Quebec) in 2006, Afluria^®^ (bioCSL) in 2007, Agriflu^®^ in 2009 (Novartis), and Fluad^®^ in 2015 (Novartis). At the present time, confirmatory studies have verified the clinical benefit for Fluarix^®^, FluLaval^®^, Afluria^®^, and Agriflu^®^.

## Annual update of seasonal influenza vaccines

To maintain effectiveness, the composition of seasonal influenza vaccines must be reviewed and updated periodically to include the most current HA antigens expressed by circulating influenza wild‐type viruses. This is a complex, lengthy process that requires extensive collaboration among influenza manufacturers, vaccine regulators, and global public health laboratories. The process begins with the recommendations, coordinated globally by the World Health Organization (WHO), for the virus strains to be included in the vaccine.^4^ The WHO recommendations for the Northern Hemisphere, which are based on recent global surveillance data available each February, provide a guide to national public health authorities and vaccine manufacturers for the development and production of influenza vaccines for the following winter influenza season. However, as noted in the WHO recommendations, it is the responsibility of each national regulatory authority to approve the composition and formulation of the vaccines used in that country. Each year, soon after the WHO Northern Hemisphere vaccine recommendations are finalized, the FDA convenes its Vaccines and Related Biological Products Advisory Committee (VRBPAC), typically in late February or early March, to recommend the virus strains that should be included in FDA‐licensed influenza vaccines for the next winter influenza season in the United States.

**Figure 1:**
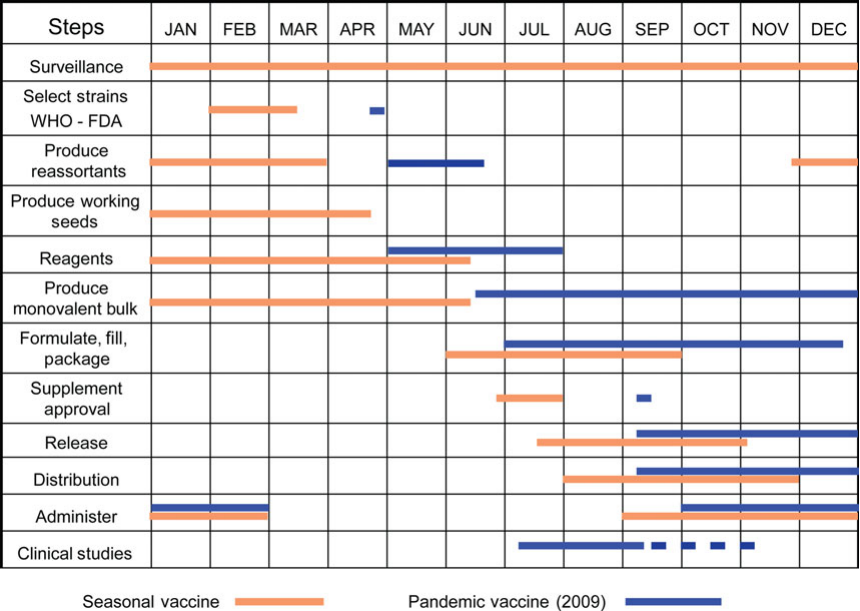
Timelines of influenza vaccine production. The various activities associated with the annual production of influenza vaccine in the Northern Hemisphere are listed and the approximate times during the year when they take place are shown. For comparison, the timing of these activities during the 2009 H1N1 pandemic is overlaid in blue.

In the United States, licensed influenza vaccine manufacturers must submit a supplement to their license for review and obtain FDA approval before the updated version of the influenza vaccine containing new virus antigens can be distributed. Such supplements to inactivated and recombinant protein seasonal influenza vaccines do not require additional clinical data specific for the new strain. Supplements to the licensed live influenza virus vaccine require a study in approximately 300 adults prior to approval of the new strain to verify adequate attenuation. Manufacturing of influenza vaccine takes place over an approximately 6‐month time frame beginning prior to the VRBPAC strain selection and lasting until mid‐summer, when the trivalent or quadrivalent vaccine is formulated, filled, and distributed. The manufacturing timelines are tight and the process of producing influenza vaccine involves many sequential steps and overlapping processes. Even with technologic advancements, each of these steps and processes still requires time to complete, and there is limited flexibility in the timelines for manufacturing. A schematic diagram of the process and key steps is shown in Figure 1.

Candidate vaccine viruses, which have been adapted for high growth in embryonated eggs and verified by a WHO Collaborating Center as antigenically like the recommended vaccine strain, are provided to manufacturers to generate “seed viruses” for inactivated seasonal influenza vaccine production. The availability of a “high‐yield” candidate vaccine virus is a key step in manufacturing, because poorly growing viruses extend the time needed for producing a sufficient amount of vaccine antigen. Manufacturers' “seed viruses,” which have been passed several more times to improve growth in their production systems, are tested by the FDA in an HI assay to ensure they remain antigenically similar to the recommended vaccine strain. Candidate vaccine viruses used for manufacturing live attenuated influenza vaccines conform to the same WHO/VRBPAC recommendations, but are tested for antigenic identity at the WHO Collaborating Center at the CDC.

Production of each vaccine antigen to be included in the vaccine takes place sequentially over several months until late May, and in fact, production of at least one antigen usually begins at risk before the strain recommendations are finalized. In parallel with antigen production, FDA develops and calibrates reagents that are necessary for testing the potency and identity of each of the antigens included in inactivated and recombinant vaccines. The reagents are provided to the vaccine manufacturers and used by both manufacturers and FDA for testing, quality control, and release of the new formulation of the licensed seasonal influenza vaccines. Standardized reagents ensure that inactivated vaccines produced by multiple manufacturers contain the same amount of HA antigen for each of the recommended virus strains. During the vaccine production process, manufacturers of inactivated and recombinant influenza vaccines submit 3‐5 lots of monovalent vaccine produced for each strain for concurrence potency testing by the FDA. This process assures harmonization of the potency assay and is designed to minimize the chances of discrepancies between the potency assigned by the manufacturer and lot release testing done by the FDA for the final formulated vaccine that could result in delay of vaccine distribution. Although preparation of potency reagents has not delayed the production and availability of seasonal influenza vaccines in the United States, timely reagent production is challenging and always a potential bottleneck in influenza vaccine production.^5^

When all of the antigens that will constitute the trivalent or quadrivalent influenza vaccine have been produced, they are blended and the vaccine is formulated into standard dosages, filled, and finished by the manufacturers into final containers such as vials, syringes, and sprayers. Manufacturers submit their vaccine testing results, along with samples from each lot, to FDA for “lot release.” Typically, FDA approves the updated seasonal influenza vaccines with new labeling by the end of July, and as FDA releases specific lots, the manufacturers make these lots commercially available throughout the United States. The dating period, or expiry, of the vaccine is based on stability studies conducted by each manufacturer, but no final vaccine formulations in the United States are labeled with an expiry date that extends past June 30 of the prior winter influenza season. This chosen expiry date is well past the Northern Hemisphere influenza season and serves to avoid any possible confusion with the influenza vaccines produced for the subsequent influenza season.

## Licensure of monovalent seasonal influenza vaccines

Prior to 1978, inactivated seasonal influenza vaccines were monovalent and bivalent; since 1978, most US licensed influenza vaccines have been trivalent incorporating two influenza A subtype viruses (H1N1 and H3N2) and an influenza B virus. More recently, quadrivalent inactivated influenza vaccines containing an additional influenza B virus antigen have been licensed. However, monovalent seasonal influenza vaccines have also been licensed as supplemental vaccines to a manufacturer's existing influenza vaccine license. For example, in 1986, a newly emerged antigenic variant of influenza A H1N1, A/Taiwan/1/86, began to circulate among the human population, which had little prior immunity, and this virus was poorly inhibited by antibodies induced by previously circulating H1N1 strains.^6^ Because of the novel characteristics of this H1N1 virus, a monovalent influenza A/Taiwan/1/86 H1N1 was approved as a supplemental vaccine to each manufacturer's license application and recommended for use.^7^ As discussed below, a similar regulatory approach was used in 2009 in response to the emergence of the 2009 H1N1 pandemic virus.

## Licensing of pandemic influenza vaccines

An effective and timely vaccine response to the emergence of a novel influenza virus into the human population will be extremely challenging. Major issues include risk assessment of the new virus threat, generation of a viable candidate vaccine virus, large‐scale vaccine manufacturing and possible clinical evaluation, and regulatory approval of the new vaccines. In the United States, there are several possible regulatory pathways to facilitate pandemic influenza vaccine availability, including vaccine use as an investigational new drug (IND), use under emergency use authorization (EUA), and use as a licensed vaccine. The regulatory mechanism that can be utilized for a particular pandemic influenza vaccine is governed by the amount and interpretability of clinical data available, and each mechanism has implications for vaccine uptake and availability. During the 2009 H1N1 influenza pandemic, licensed influenza vaccine manufacturers in the United States produced monovalent vaccines containing this novel H1N1 virus strain that were approved as supplements to their seasonal license, essentially using the same strain change supplement process for seasonal vaccines described above. Although the swine‐origin H1N1 virus that triggered the pandemic was unique, the H1N1 subtype was not novel, and this regulatory pathway was consistent with prior regulatory actions by the FDA such as the monovalent H1N1 (A/Taiwan/1/1986) supplemental vaccine produced in 1986. The decision to consider this updated H1N1 vaccine as a strain change to a licensed vaccine meant that clinical trials to determine dose and schedule of the vaccine were not needed for licensure, and this undoubtedly shortened the time to vaccine availability (Figure 1). Nevertheless, the bulk of monovalent 2009 H1N1 pandemic vaccine became available only after the peak of the H1N1 infections in September–November 2009.

In contrast to the 2009 H1N1 pandemic, where clinical trials that were conducted confirmed that the typical seasonal dose of H1N1 vaccine was sufficient to induce a protective antibody response, data from clinical trials with novel influenza pandemic subtypes (e.g., H5N1, H7N9) indicate that a seasonal vaccine dose is unlikely to be successful for all influenza strains.^8^, ^9^, ^10^, ^11^ Thus, clinical data are needed to make informed decisions about vaccine dose and schedule, as well as to provide safety data, for novel influenza subtypes.

**Figure 2:**
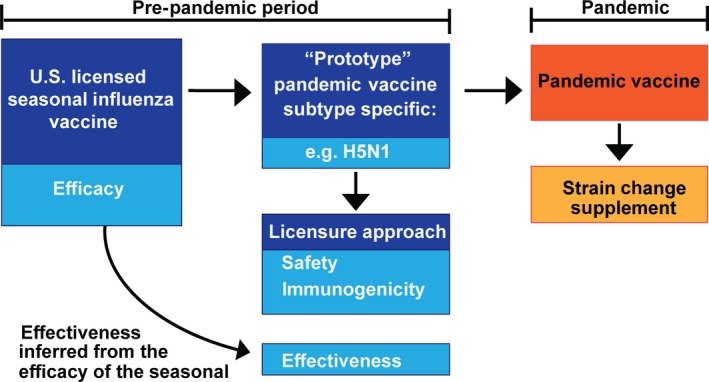
Licensing of pandemic influenza vaccines. A schematic diagram for licensing of pandemic influenza vaccines. The effectiveness of a pandemic influenza vaccine is inferred from the demonstrated efficacy of a licensed seasonal vaccine made by the same manufacturing process and supported by clinical studies supporting the safety and immunogenicity of the pandemic vaccine. During a pandemic, the license can be updated with a strain change of the same HA subtype in a process similar to that used for updating seasonal vaccines.

Rapid regulatory approval and vaccine availability for such novel pandemic influenza virus would be facilitated by prior licensure of a vaccine consisting of the novel pandemic subtype, referred to as the prototype, so that in the event of a pandemic, a better matched candidate vaccine virus could be substituted as a strain change supplement to the license as described above for seasonal vaccines. A schematic diagram of the regulatory path for licensure of pandemic influenza vaccines is shown in Figure 2. The general approach has been described previously,^12^ and there has been additional public discussion about this regulatory approach for specific pandemic vaccines with the FDA's VRBPAC. To date, two pandemic influenza vaccines have been licensed in the United States, an H5N1 vaccine manufactured by Sanofi Pasteur and an adjuvanted H5N1 vaccine manufactured by ID Biomedical Corporation of Quebec (GSK) containing AS03 (an oil‐in‐water emulsion adjuvant with α‐tocopherol and squalene 13). In both situations, clinical trials were conducted with prototype pandemic vaccines to establish safety and immunogenicity of the selected dose and schedule. Effectiveness of the vaccine was inferred from the known efficacy of the seasonal vaccine manufactured by the same manufacturer, using the same process. While several regulatory mechanisms exist to make vaccine available during a pandemic and would certainly be used as needed, a licensing strategy with clinical trials conducted during the pre‐pandemic period offers a clear and relatively straightforward pathway for implementation of a strain‐specific pandemic vaccine.

## Regulatory experience and challenges with new and novel influenza vaccines

As noted earlier, current influenza vaccines are neither rapidly produced, nor ideal in terms of protection. Consequently, significant effort has been expended toward developing and approving improved vaccines, for example, development of quadrivalent vaccines containing antigens from two influenza B virus lineages, cell culture‐produced vaccines, recombinant protein‐based vaccines, and adjuvanted vaccines.

The need for a quadrivalent vaccine containing two influenza B antigens was driven by the emergence since the mid‐1980s of two antigenically distinct lineages of influenza B that co‐circulate.^14^ Although public health agencies continued to recommend the influenza B lineage thought most likely to prevail in the upcoming influenza season, the predominant circulating strain often was different from the recommended strain, indicating a need for development of a vaccine that would provide protection against both types of influenza B.^15^, ^16^ At the present time, four quadrivalent influenza vaccines have been licensed in the United States: FluMist Quadrivalent^®^ (MedImmune—2012), Fluarix Quadrivalent^®^ (GSK – 2012), Fluzone Quadrivalent^®^ (Sanofi Pasteur—2013), and FluLaval Quadrivalent^®^ (IDB—2013). In each case, the quadrivalent vaccine was approved as part of the existing seasonal license by submission of a supplement providing clinical data for safety and immunogenicity that demonstrated that addition of a fourth component to the vaccine did not result in an inferior immune response to the other vaccine components compared to the existing trivalent vaccine. The process for selecting the 2nd influenza B strain for the vaccine is similar to that used for selection of the other strains of the vaccine as described above. The data‐driven WHO and VRBPAC strain‐selection process provide a recommendation for the influenza B strain to be included in trivalent vaccines, as well as a recommendation for the 2nd influenza B strain to be included in quadrivalent vaccines. Ongoing challenges in the regulation of quadrivalent influenza vaccines include the preparation of a 4th set of potency reagents within the tight timeline of vaccine production, and the potential for cross‐reactivity between the reagents developed for identity and potency testing of the two influenza B antigens in the vaccine.

While the majority of influenza vaccines available in the United States are still produced in embryonated chicken eggs, one cell‐based influenza vaccine was licensed in 2012 (Flucelvax^®^). Cell‐based vaccines have several potential advantages compared to egg‐based vaccines, including the possibility that propagation in cell culture may avoid common egg‐adapted changes in HA that can sometimes lead to antigenic changes.^17^ Flucelvax was approved as a new vaccine based on demonstration of safety and efficacy in pre‐licensure clinical trials. Approval followed extensive discussions with the advisory committee that affirmed the safety of MDCK cells used as the substrate for vaccine production. MDCK cells are a continuous cell line derived from a dog kidney and known to be tumorigenic in nude mice. The selection of vaccine strains for cell‐based influenza vaccines is the same as for egg‐based vaccines, following WHO and VRBPAC recommendations. At the time of licensure, Flucelvax^®^ was approved to use the same candidate vaccine viruses, characterized, and verified by WHO Collaborating Centers that are used for egg‐based influenza vaccines. By convention, and based on extensive experience demonstrating their suitability and safety, current candidate vaccine viruses are originally derived by isolation in embryonated eggs. It is likely that additional cell‐based influenza vaccines will be developed and there are several regulatory challenges that will need to be confronted. For example, egg‐adaptation and generation of high growth reassortants suitable for vaccine manufacture in egg systems may offer little advantage to a cell‐based production process, but to date there is no common established approach that explicates how and under what conditions a virus originally isolated in cell culture can be used as a candidate vaccine virus. Cell‐based vaccine manufacturers are encouraged to work with the FDA to address this issue and related issues such as those related to potential adventitious agent contamination due to the use of mammalian cells for virus isolation, and the possible amplification of such agents in a cell culture system. In addition, cell‐based influenza vaccines present additional challenges for reagent production by regulatory agencies. The current working model is to prepare specific cell‐based reference antigen for cell‐based vaccines, but continue the use of a common potency antiserum that generally has used an egg‐derived HA antigen for immunization. The need for dedicated reagents for cell‐based vaccine systems presents additional resource issues and further stresses on the seasonal timelines that can only be expected to increase as other cell‐based systems, using other cell lines, are developed.

A recombinant protein influenza vaccine, Flublok^®^, was licensed in 2013. This was the first trivalent influenza vaccine made using an insect virus (baculovirus) expression system and recombinant DNA technology. As noted earlier, licensure was based on safety and efficacy assessed in pre‐licensure clinical trials, and the selection of strains follows the usual WHO and VRBPAC process. Unlike other current licensed influenza vaccines, recombinant protein vaccines do not require candidate vaccine viruses, and development of an updated vaccine can begin with HA sequence information. In addition, like mammalian cell culture processes, the scale‐up potential of the insect cell/baculovirus vector system may offer advantages for rapid antigen change and response to a pandemic situation.^18^ Some of these potential advantages may also apply to other novel recombinant protein vaccines that are being developed.^19^, ^20^ Regulatory challenges for novel influenza vaccines include the determination of the most appropriate potency assay for a particular vaccine product. The single radial immunodiffusion (SRID) assay established for egg‐based inactivated vaccines may not be optimal, or even appropriate, for novel types of influenza vaccines that are fundamentally different from the traditional egg‐based inactivated vaccines. In addition, the ease of nucleic acid manipulation offers the possibility of product improvement through relatively rapid generation of recombinant derivatives. The regulatory pathways for when and how such vaccine derivatives can be integrated into the existing license are not yet established.

Adjuvanted influenza vaccines have been proposed as offering many potential improvements to inactivated influenza vaccines, including, among others, the possibilities of dose‐sparing, broadening the immune response to heterotypic antigens, and enhancing the immune response in certain populations such as children and the elderly.^21^ To date, there are 2 licensed adjuvanted influenza vaccines in the United States, a pandemic H5N1 vaccine combined with AS03 noted earlier, and Fluad^®^ (Novartis),^22^ a seasonal trivalent inactivated influenza vaccine combined with MF59C.1 (a squalene‐containing oil‐in‐water emulsion adjuvant) for immunization of adults 65 years and older that was licensed in 2015. Fluad^®^ was licensed using the accelerated approval mechanism described earlier, so a confirmatory efficacy trial is required to verify and describe the clinical benefit. In general, the regulatory pathways supporting the development and approval of vaccines formulated with novel adjuvants are the same as for unadjuvanted vaccines. Adjuvants are not licensed separately, but rather in combination with an antigen as an adjuvanted vaccine. However, adjuvants exhibit a range of properties that invoke complex immune responses and their mechanism of action is not always known or fully understood. Further, animal models that predict the safety and efficacy of an adjuvant–antigen complex are usually not available. Consequently, an efficient development pathway for an adjuvanted vaccine requires careful attention to pre‐clinical testing, study design, dosing decisions, and safety monitoring. Although manufacturers are not required to demonstrate the added benefit of an adjuvanted vaccine compared to a non‐adjuvanted vaccine in pivotal phase 3 efficacy studies, justification for including an adjuvant in the vaccine is needed, and evaluation of the safety of an adjuvanted vaccine will include special safety considerations. Such considerations may include an extended duration of safety follow‐up, as well as monitoring of special interest adverse events and potential autoimmune/auto‐inflammatory events.

## Summary

In summary, the regulation of influenza vaccines is unique and challenging, as is the manufacture of influenza vaccines. In spite of the remaining challenges to improve influenza vaccines, substantial progress has been made over the past few years. In the United States, over 150 million doses of seasonal trivalent and quadrivalent vaccine are produced annually, a mammoth effort, particularly in the context of a vaccine with components that usually change on a yearly basis. Reagents for quantifying the antigen content of inactivated vaccines and ensuring standardization of vaccines made by different manufacturers are produced and distributed routinely without delaying vaccine availability. Pandemic vaccines have been licensed, and the pathways for licensure of pandemic vaccines and subsequent strain updating have been defined. Complex issues confronted during evaluation of novel influenza vaccines have been resolved satisfactorily with the result that new types of influenza vaccines, including cell‐based vaccines, recombinant protein vaccines, and adjuvanted vaccines, are now available for use. Having such a variety of types of influenza virus vaccines is important for the flexibility needed to generate a timely vaccine response to the ever changing nature of influenza viruses, both unexpected drifted virus strains and the emergence of new influenza subtypes.

While challenges to implementation of better and more rapid production of influenza vaccines remain, extensive effort has been made over the past few years to explore strategies that address potential bottlenecks in the manufacturing process, as well as new approaches that might speed‐up other parts of the influenza vaccine process. Examples of such efforts include the following:

- Improvement of the global strain‐selection process, coordinated by WHO, to incorporate newer methods for predicting virus evolution and drift and identification of virus vaccine candidates 23, 24
- Increasing the availability of more high‐yield candidate vaccine viruses, including candidate vaccines that are optimized for specific production systems (e.g., cell‐based or egg production) 25, 26
- Coordinating reagent production among regulatory agencies to address resource issues and developing backup strategies for reagent production to ensure availability in the case of unexpected problems
- Accelerating the process of potency reagent calibration and developing improved methods for vaccine testing, such as new methods for testing potency and sterility
- Developing new types of influenza vaccines with greater cross‐protection and longer duration of protection
- Identifying issues related to development and evaluation of novel influenza vaccines so that appropriate regulatory pathways can be employed to facilitate licensure

These, as well as other unforeseen issues and problems, will be challenging for regulatory agencies as well as vaccine manufacturers. Nevertheless, the history of collaborative interactions among all of the global partners in the influenza vaccine endeavor lays a strong foundation for continued progress.
